# PD-L1 expression in EBV associated gastric cancer: a systematic review and meta-analysis

**DOI:** 10.1007/s12672-022-00479-0

**Published:** 2022-03-22

**Authors:** Áurea Lima, Hugo Sousa, Rui Medeiros, Amanda Nobre, Manuela Machado

**Affiliations:** 1 Serviço de Oncologia Médica do Centro Hospitalar de Entre o Douro e Vouga, Unidade de Santa Maria da Feira, Rua Dr. Cândido Pinho 5, 4520-211 Santa Maria da Feira, Portugal; 2 CESPU, Instituto de Investigação e Formação Avançada em Ciências e Tecnologias da Saúde (IINFACTS), Rua Central de Gandra 1317, 4585-116 Gandra PRD, Portugal; 3 Molecular Oncology and Viral Pathology Group, Research Center of IPO Porto (CI-IPOP)/RISE@CI-IPOP (Health Research Network), Portuguese Oncology Institute of Porto (IPO Porto)/Porto Comprehensive Cancer Center (Porto.CCC), Rua Dr. António Bernardino de Almeida, 4200-072 Porto, Portugal; 4 Serviço de Virologia, Instituto Português de Oncologia do Porto FG EPE (IPO Porto), Rua Dr. António Bernardino de Almeida, 4200-072 Porto, Portugal; 5 Early Phase Clinical Trials Unit – Clinical Research Unit &/RISE@CI-IPOP (Health Research Network), Portuguese Oncology Institute of Porto (IPO Porto)/Porto Comprehensive Cancer Center (Porto.CCC), Rua Dr. António Bernardino de Almeida, 4200-072 Porto, Portugal

**Keywords:** Gastric cancer, PD-L1, EBV, Microsatellite instability, Immunotherapy, GCLS

## Abstract

**Objectives:**

The aim of this systematic review and meta-analysis is to the summarize the evidence on programmed cell death protein ligand 1 (PD-L1) in Epstein-Barr virus associated gastric cancer (EBVaGC) and to estimate the expression rate of PD-L1 among this subtype of Gastric Cancer (GC).

**Materials and methods:**

For this study, PubMed^®^, EMBASE^®^ and Web of Science^®^ databases were searched for articles published until 1st November 2021. A total of 43 eligible publications with a total of 11,327 patients were included analysis based on inclusion and exclusion criteria. A total of 41 publications present data for proportion estimation and 33 for comparison of PD-L1 between EBV positive and negative GC. DerSimonian-Laird random-effects model was used for meta-analysis.

**Results:**

The analysis showed that in EBVaGC the pooled positivity rate for PD-L1 was 54.6% (*p* < 0.001), with a high heterogeneity between the included studies, which was associated with variation on positivity criteria for PD-L1 expression. Overall, the study reveals an increased association between PD-L1 and EBVaGC (OR = 6.36, 95% CI 3.91–10.3, *p* < 0.001). Furthermore, the study revealed that GC with lymphoid stroma (GCLS) is highly associated with EBV (OR = 17.4, 95% CI 6.83–44.1, *p* < 0.001), with a pooled EBV positivity rate of 52.9% (*p* < 0.001).

**Conclusions:**

Patients with EBVaGC tend to show higher PD-L1 expression, which enhances EBV positivity as a promising marker for patient selection for immunotherapy targeted agents. A uniform criteria for PD-L1 positivity in tumor cells is needed, as well as further prospective studies to validate our findings and their prognostic significance.

**Supplementary Information:**

The online version contains supplementary material available at 10.1007/s12672-022-00479-0.

## Introduction

Gastric Cancer (GC) is the fourth most deadly and sixth most incident malignant tumor worldwide, with more than a million new cases in 2020 [1]. In 2014, *The Cancer Genome Atlas* (TCGA) group proposed a classification of GC into four distinct subtypes: (1) microsatellite unstable tumors (MSI); (2) genomic stable tumors (GS); (3) tumors with chromosomal instability (CIS); and (4) tumors positive for Epstein–Barr Virus (EBVaGC) [2, 3].

Nowadays, it is widely accepted that EBVaGC represents almost 10% of all GC [4–7]. This subtype has distinctive pathologic and genomic profiles. Pathologically, EBVaGC is often usually found in the proximal stomach, and is characterized by a moderate to poor degree of differentiation and shows better prognosis than EBV-negative GC [8–13]. The genomic profile of EBVaGC reveals an extensive CpG island methylation, higher levels of programmed death ligands 1 and 2 (PD-L1/2), different PIK3CA mutation pattern and no p53 mutations are observed [2, 10, 14–19]. EBV is also present in over 80% of GC with lymphoid stroma (GCLS) cases, a particularly rare histological type of GC [20, 21].

Most patients with GC are diagnosed at advanced stages of disease, which has a significant impact on the potential for successful treatment. Primary surgical resection with adjuvant chemotherapy or chemoradiotherapy or perioperative chemotherapy are the main treatment strategies for gastric cancer but, unfortunately, only a modest survival advantage is obtained for patients with advanced GC despite significant effort in both clinical and preclinical research. The identification of novel therapeutics for the treatment of advanced GC represents an important area of investigation [3, 22–25]. Over the last decade, the better understanding of immune checkpoints in cancer development, prompted the appearance of novel immunotherapy agents like programmed cell death 1 (PD-1) and programmed death-ligand 1 (PD-L1) inhibitors which demonstrated to be surprisingly effective in the treatment of different types of cancer [26, 27].

PD-1 is a transmembrane protein, highly expressed in tumor specific T-cells, that inhibits both innate and adaptative response. PD-1 interacts with PD-L1, a transmembrane glycoprotein, usually expressed in immune, dendritic and epithelial cells, and that can also be expressed by some tumor cells [28]. Pembrolizumab, an anti-PD-1 antibody, was the first agent to be approved by the United States *Food and Drug Administration* (FDA) in a non-primary tumor dependent manner, as second line treatment for metastatic or unresectable solid tumors showing high microsatellite instability (MSI-H) or deficient mismatch repair (MMR) [29]. It was specifically approved for recurrent and metastatic GC following two or more lines of therapy, after the results from KEYNOTE-012 trial showing a promising overall response rate [30]. The phase II clinical trial KEYNOTE-059 confirmed the efficacy of pembrolizumab in monotherapy as a third line for GC presenting a combined positive score (CPS) ≥ 1 [31]. Hence, considering that PD-L1 overexpression has been widely described for EBVaGC [32, 33], there are already some clinical trials ongoing testing anti-PD-1 drugs such as nivolumab (NCT02951091) or avelumab (NCT01772004) in with EBVaGC [34–36].

Although EBV and PD-L1 expression are both associated with GC, there is conflicting evidence on the association of both. Through a systematic review and meta-analysis, we aim to assess whether there is evidence on the higher expression rate of PD-L1 in EBV positive GC and to estimate the expression rate of PD-L1 among this specific subgroup. Furthermore, we intend to evaluate if there is evidence for a higher rate of EBV positive or PD-L1 expression in GCLS.

## Material and methods

### Literature search and study selection

A systematic review of the literature was performed using the Preferred Reporting Items for Systematic Reviews and Meta-Analyses (PRISMA) guidelines. The literature search was performed in both PubMed^®^, EMBASE^®^ and Web of Science^®^ databases on the 1st of November 2021 using a combination of controlled terms (MeSH and EMTREE) and synonyms. The following MeSH terms were used: “Stomach Neoplasms”[Mesh], “CD274 protein, human”[Supplementary Concept], : “Herpesvirus 4, Human”[Mesh]; the correspondent EMTREE terms were as follows: ‘stomach cancer’, ‘pd l1 protein’, ‘epstein barr virus’. The literature search was performed independently by two of the authors (AL and HS) with no restriction on time, sample size or population.

The resulting search queries according to each database were, for PubMed^®^ (“Stomach Neoplasms”[Mesh] OR “Gastric cancer” OR “Gastric cancers” OR “Gastric Neoplasm” OR “Gastric Neoplasms” OR “Stomach Cancer” OR “Stomach Cancers” OR (gastric AND (cancer OR neoplasm))) AND (“CD274 protein, human” [Supplementary Concept] OR “B7-H1 Antigen”[Mesh] OR PD-L1 OR “Programmed death-ligand 1” OR “Programmed death ligand 1” OR “Programmed Cell Death 1 Ligand 1”) AND (“Herpesvirus 4, Human”[Mesh] OR EBV OR “Epstein-Barr” OR “Epstein-Barr Virus” OR “Epstein Barr Virus” OR “HHV-4” OR “Human Herpesvirus 4”); for EMBASE^®^ (‘stomach cancer’/exp OR ‘stomach cancer’ OR ‘gastric cancer’ OR ‘stomach cancers’ OR ‘gastric cancers’ OR ‘stomach tumor’/exp OR ‘stomach tumor’) AND (‘pd l1 protein’/exp OR ‘pd l1 protein’ OR ‘pd l1’ OR ‘programmed death ligand 1’ OR ‘programmed death-ligand 1’ OR ‘b7 h1’ OR cd274) AND (‘epstein barr virus’/exp OR ‘epstein barr virus’ OR ebv OR ‘herpesvirus 4’ OR ‘hhv 4’); and for Web of Science^®^ (“Stomach Neoplasms” OR “gastric cancer” OR (gastric AND (cancer OR neoplasm)) OR “stomach cancer”) AND (cd274 OR b7-h1 OR pd-l1 OR “Programmed death-ligand 1” OR “Programmed death ligand 1”) AND (“Herpesvirus 4 ” OR EBV OR “Epstein-Barr” OR “Epstein Barr” OR “Epstein-Barr Virus” OR “Epstein Barr Virus” OR HHV-4).

The following inclusion criteria were considered: (1) histologically confirmed GC; (2) histological characterization; (3) age > 18 years old; (4) EBV status information; and (5) PD-L1 analysis (independently of the method). Studies were excluded if: (1) written in other languages than English; (2) duplicated data; (3) other study design (case reports, comments, series, reviews, and editorials); and (4) insufficient data or data not available. All review studies were checked for their references for other relevant studies. The reference lists of the selected studies were also reviewed and compared with our list of included studies.

### Data extraction

According to PRISMA guidelines, each step was performed independently by two investigators and discrepancies were decided by a third investigator. Briefly, manuscripts were first screened by analyzing titles and abstracts, based on the inclusion/exclusion criteria. Full texts were then reviewed, and data extracted (first author, year of publication, original country, number of cases, age, gender, staging, histological type, EBV status and PD-L1 expression). A qualitative analysis was performed based on the Newcastle–Ottawa Scale (NOS) for case–control studies [37]. All articles with a score above 8 were considered high-quality studies.

### Statistical analysis

Meta-analysis for comparison of PD-L1 expression between EBV associated GC and EBV negative GC was performed using the open-source software jamovi, version 1.6.23, using the METAFOR package [38–40]. All studies that described PD-L1 expression in both EBV positive and EBV negative GC were included in the meta-analysis. Estimates of odds ratio (OR) were weighted and pooled according to the DerSimonian-Laird random-effects model. An OR > 1.00 represents a higher expression of PD-L1 in EBV positive GC, while an OR < 1.00 describes a higher expression in EBV negative GC. Also, all studies reporting the rate of PD-L1 positivity among EBV positive GC were included in a meta-analysis for proportions, in order to estimate its pooled rate. Furthermore, all studies specifically mentioning GCLS histology were assessed in a meta-analysis to determine the pooled proportion of EBV and PD-L1 positivity among this histology. Heterogeneity between studies was assessed by Cochrane Q-test and I^2^ determination and publication bias was evaluated using a funnel-plot approach and its asymmetry tested using a regression test, for all meta-analysis. A *p*-value less than 0.05 was considered statistically significant.

## Results

### Characteristics of included studies

**Fig. 1:**
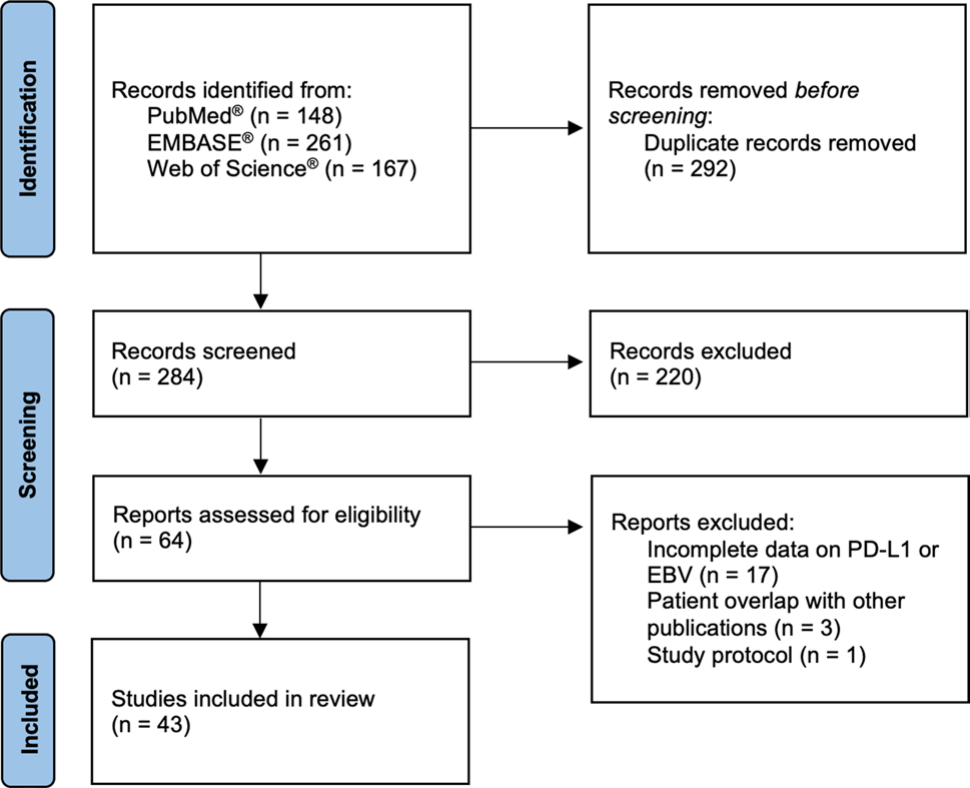
PRISMA flow chart for systematic review and meta-analysis

The study selection flow diagram is presented in Fig. 1 The literature search in PubMed^®^ provided a total of 148 manuscripts, while search in EMBASE^®^ showed 261 results and in Web of Science^®^ a total of 167 publications. After duplicate removal, a total of 284 records were screened by title and abstract, with a total of 220 articles excluded due to the following reasons: in vitro studies, letter to the editor, other tumor locations, no assessment/reporting on EBV or PD-L1 expression, case reports, trial protocol, review articles and meta-analysis. A total of 64 manuscripts were assessed for full review, with exclusion of 21 studies due to incomplete data (n = 17), overlap of patients with other included publications (n = 3) and reporting a study protocol (n = 1). The analysis result in the inclusion of a total of 43 publications [19, 41–82]. Among the included studies, we observed that 28 studies were performed in Asian populations, 6 in Europe, 6 in North America and the remaining in Brazil and Morocco.

### Study and patient characteristics

Table 1 summarizes the characteristics of the 43 included publications, comprehending a total of 11,327 patients, ranging from 9 to 1000 participants. One publication had two patient sets, namely an experimental (273 cases) and a validation set (159 cases) [53]. The majority of patients were over 60 years old in most studies, with a male predominance and patients undergoing surgical resection of the primary tumor in most of these sets. Thirteen studies did not include patients with metastatic disease, [43, 45, 46, 53, 54, 56, 57, 59, 60, 66, 70, 74, 80] while 2 studies included only metastatic GC patients [50, 52] and 1 did not include early stage GC cases [69].

**Table 1:** Characteristics of the studies included in the systematic review

Authors (reference number), year	Country	Total patients number	Age (years)	Males (%)	Stage	Histology	EBV+ (%)	PD-L1 expression in TC (EBV+, %)	PD-L1 positivity criteria
Moreira-Nunes *et al*. [41], 2021	Brazil	1000	≥ 64: 382 (38.2%)	658 (65.8%)	I–IV	Diffuse: 412 (41.2%) Intestinal: 588 (58.8%)	190 (19.0%)	149 (78.4%)	Comparison to non-tumor controls (higher vs. lower)
Nshizirungu *et al.*[42], 2021	Morocco	97	Mean: 59	59 (60.8%)	NA	Diffuse: 32 (32.9%) Intestinal: 65 (67.1%)	6 (6.2%)	1 (33.3%) Total of 3 EBV+ cases	CPS > 1
Yang *et al.* [43], 2021	China	226	≥ 60: 134 (59.3%)	172 (76.1%)	I–III	Tubular: 165 (73.0%) Mucoid: 28 (12.4%) Signet-ring cell: 30 (13.3%) Other: 3 (1.3%)	13 (5.8%)	9 (81.8%) Total of 11 EBV+ cases	IRS > 2
Choi *et al*. [44], 2020	Korea	514	Median: 65	347 (67.5%)	I–IV	Diffuse: 228 (44.4%) Intestinal: 286 (55.6%)	32 (6.2%)	15 (46.9%)	Any membrane staining in tumor cells
Di Pinto *et al*. [45], 2020	Italy	70	Median: 65.8	46 (65.7%)	I–III	Diffuse: 36 (51.4%) Intestinal: 34 (48.6%)	2 (2.9%)	2 (100%)	≥ 5% tumor cells with membrane staining
Fang *et al*. [46], 2020	Taiwan	460	≥ 65: 276 (60.0%)	329 (71.5%)	I–III	GCSL: 30 (6.5%) Diffuse: 212 (46.1%) Intestinal: 218 (47.4%)	43 (9.3%)	20 (46.5%)	CPS ≥ 1
Hyun Kim *et al*. [47], 2020	Korea	286	Mean: 60.8	187 (65.4%)	I–IV	Diffuse: 73 (25.5%) Intestinal: 176 (61.5%) Mixed: 37 (12.9%)	17 (5.9%)	10 (58.8%)	≥ 1% tumor cells with membrane staining
Liu *et al*. [48], 2020	Korea	300	≥ 64: 152 (50.7%)	199 (66.3%)	I–IV	Diffuse: 150 (50.0%) Intestinal: 142 (47.3%) Mixed: 8 (2.7%)	18 (6.5%) Total of 275 cases	17 (94.4%)	CPS ≥ 1
Martinson *et al*. [49], 2020	USA	85	Median: 60.9	52 (61.2%)	I–IV	Diffuse: 44 (51.8%) Intestinal: 41 (48.2%)	19 (22.4%)	7 (36.8%)	CPS ≥ 1
Xie *et al*. [50], 2020	China	9	Mean: 60.7	8 (88.9%)	IV	Adenocarcinoma: 8 (88.9%)Signet-ring cell carcinoma: 1 (11.1%)	9 (100%)	7 (77.8%)	≥ 5% tumor cells with membrane staining
Gullo* et al.* [51], 2019	Portugal	78	> 60: 61 (78.2%)	45 (57.7%)	I–IV	GCLS: 24 (30.8%)	19 (24.4%)	NA	IRS > 2
Kawazoe *et al.* [52], 2019	Japan	225	Median: 66	136 (60.4%)	IV	Diffuse: 155 (68.9%) Intestinal: 70 (31.1%)	14 (6.2%)	3 (21.4%)	≥ 1% tumor cells with membrane staining
Kim YB* et al.* [53], 2019,	Korea	432							
Experimental set		273	Mean: 58.7	190 (69.6%)	I–III	Diffuse: 110 (40.3%) Intestinal: 149 (54.6%) Mixed: 14 (5.1%)	25 (9.1%)	10 (40.0%)	≥ 5% tumor cells with membrane staining
Validation set		159	Mean: 62.2	110 (69.2%)	I–III	NR	9 (5.7%)	5 (55.6%)	≥ 5% tumor cells with membrane staining
Kim JY *et al*. [54], 2019	Korea	297	Mean: 62.4	204 (68.7%)	I–III	Diffuse: 118 (39.7%) Intestinal: 130 (43.8%)| Mixed: 49 (16.5%)	22 (7.4%)	4 (18.2%)	> 5% tumor cells with membrane staining
Mishima *et al*. [55], 2019	Japan	80	Median: 67	61 (76.3%)	I–IV	Diffuse: 46 (57.5%) Intestinal: 34 (42.5%)	4 (5.0%)	0 (0%)	≥ 5% tumor cells with membrane staining
Nakayama *et al*. [56], 2019	Japan	43	> 65: 23 (53.5%)	31 (72.1%)	I–III	Diffuse: 14 (32.6%) Intestinal: 29 (67.4%)	43 (100%)	15 (71.4%)	≥ 5% tumor cells with membrane staining
Setia *et al*, [57], 2019	Korea (and USA)	486	67.5±13.35	311 (64.0%)	I–III	GCLS: 17 (3.5%) Diffuse: 71 (14.5%) Intestinal: 311 (63.9%)	33 (6.8%)	4 (57.1%)*	Any membrane staining in tumor or macrophages
Sun *et al*. [58], 2019	China	165	Median: 64	117 (70.9%)	I–IV	Diffuse: 78 (47.3%) Intestinal: 70 (42.4%) Mixed: 17 (10.3%)	2 (1.2%)	1 (50.0%)	≥ 1% tumor cells with membrane staining
Valentini *et al*. [59] 2019	Italy	70	Mean: 65.83 ± 10.63	46 (66.0%)	I–III	Diffuse: 36 (51.0%) Intestinal: 34 (49.0%)	2 (2.9%)	2 (100%)	≥ 5% tumor cells with membrane staining
Yoon *et al*. [60], 2019	Canada	107	Range: 19–86	66 (61.7%)	I–III	Diffuse: 31 (29.0%) Intestinal/mixed: 76 (71.0%)	3 (2.8%)	2 (66.7%)	> 1% tumor cells with membrane staining
Chang *et al.* [61], 2018	Korea	241	≥ 60: 123 (51.0%)	161 (66.8%)	I–IV	Diffuse: 104 (43.2%) Intestinal: 103 (42.7%) Mixed / Undetermined: 34 (14.1%)	40 (16.6%)	23 (57.5%)	PD-L1 ratio > .136441 (automated method)
Cho* et al.* [62], 2018	Korea	58	Mean: 57.8 ± 11.7	46 (79.3%)	I–IV	GCLS: 58 (100%)	186 (86.5%) of a total of 215 GCLS	9 (31.0%)	≥ 25% tumor cells with membrane staining
de Rosa *et al*. [63], 2018	Italy	169	Mean: 67	103 (61%)	I–IV	Diffuse: 21 (12.4%) Intestinal: 118 (69.8%) Undetermined: 30 (17.8%)	33 (19.5%)	15 (45.5%)	≥ 5% membrane staining, any intensity
Gullo* et al.* [64], 2018	Portugal	46	NR	NR	NR	GCLS: 25 (54.3%)	15 (32.6%)	6 (40.0%)	IRS ≥ 2
Hissong *et al*. [65], 2018	USA	31	Mean: 70	23 (74.2%)	I–IV	GCLS: 31 (100%)	7 (22.5%)	5 (71.4%)	Any membrane staining in tumor cells
Noh *et al*. [66], 2018	Korea	479	≥ 63: 265 (55.3%)	353 (73.7%)	I–III	Diffuse: 163 (34.0%) Intestinal: 249 (52.0%) Mixed: 48 (10.0%) NA: 19 (4.0%)	36 (7.7%) Total of 468 cases	16 (44.4%)	IRS ≥ 2
Pereira* et al. *[67], 2018	Brazil	287	Mean: 61.5	168 (58.5%)	I–IV	Diffuse: 109 (38.1%) Intestinal: 136 (47.6%) Mixed: 28 (9,8%) Undetermined: 13 (4.5%)	30 (10.5%)	13 (44.8%)	≥ 1% tumor cells with membrane staining
Sundar* et al. *[68], 2018	Korea	220	NR	166 (75.5%)	I–IV	NR	71 (32.3%)	CPS>1: 60 (84.5%) CPS>5: 37 (52.1%)	CPS > 1 or > 5
Kawazoe *et al.* [69], 2017	Japan	487	Median: 66	327 (67.1%)	III–IV	Poorly differentiated: 169 (34.7%) Signet ring: 260 (53.4%) Other: 58 (11.9%)	25 (5.1%)	13 (52.0%)	≥ 1% tumor cells with membrane staining
Koh *et al*. [70], 2017	Korea	392	Median: 59	253 (64.5%)	II–III	Diffuse: 214 (54.6%) Intestinal: 146 (37.2%) Mixed: 30 (7.7%) Indeterminate: 2 (0.5%)	25 (6.4%)	23 (92.0%)	≥ 5% tumor cells with membrane staining
Kwon *et al*. [71], 2017	Korea	394	≥ 60: 236 (59.9%)	274 (69.5%)	I–IV	Diffuse: 126 (32.0%) Intestinal: 203 (51.5%) Mixed: 65 (16.5%)	26 (6.6%)	11 (42.3%)	> 10% tumor cells with membrane staining
Ma J. *et al*. [72], 2017	China	571	Median: 59	407 (71.3%)	I–IV	Adenocarcinoma: 529 (92.6%) Other: 42 (7.4%)	31 (5.4%)	13 (41.9%)	≥ 5% membranous expression were considered positive.
Saito* et al.* [73], 2017	Japan	232	NR	NR	NR	NR	96 (41.4%)	33 (34.4%)	> 5% tumor cells with membrane staining
Seo *et al*. [74], 2017	Korea	116	≥ 62: 61 (52.6%)	93 (80.2%)	I–III	Diffuse: 81 (69.8%) Intestinal: 24 (20.7%) Mixed: 11 (9.5%)	116 (100%)	57 (49.3%)	≥ 1% tumor cells with moderate or strong staining
Thompson* et al.* [75],2017	USA	34	Median: 67	18 (53%)	I–IV	Diffuse: 15 (44.1%) Intestinal: 19 (55.9%)	2 (5.9%)	1 (50%)	≥ 5% tumor cells with membrane staining
Wu *et al*. [76], 2017	China	340	> 45: 318 (93.5%)	254 (74.7%)	I–IV	Tubular: 244 (71.8%) Signet ring cell: 36 (10.6%) Other: 60 (17.6%)	17 (5.0%)	12 (70.6%)	IRS > 2
(Böger* et al. *[77], 2016	Germany	451	≥ 68: 233 (50.1%)	290 (64.3%)	I–IV	Diffuse: 145 (31.3%) Intestinal: 240 (51.7%) Mixed: 31 (6.7%) Unknown: 48 (10.3%)	20 (4.4%)	18 (90.0%)	IRS > 2
Dai* et al.* [78], 2016	China	398	≥ 60: 214 (53.8%)	304 (76.4%)	I-IV	Diffuse: 169 (42.8%) Intestinal: 226 (57.2%)	10 (11.5%) Total of 97 cases	7 (70%)	≥ 5% tumor cells with membrane staining or ≥ 1+ intensity
Derks* et al.* [19], 2016	USA	81	Mean: 67.7	52 (64.2%)	I–IV	Diffuse/Mixed: 15 (18.5%) Intestinal: 66 (81.5%)	32 (39.5%)	16 (50.0%)	≥ 5% tumor cells with membrane staining
Dong* et al. *[79], 2016	China	855	≥ 60: 413 (48.3%)	587 (68.7%)	I–IV	Diffuse: 508 (59.4%) Intestinal: 235 (27.5%) Mixed: 112 (10.1%)	59 (6.9%)	49 (92.5%)	Cut-off determined for this sample using a ROC curve
Kang* et al. *[80], 2016	Korea	234	Mean: 56	203 (86.8%)	I–III	Adenocarcinoma component: 129 (55.1%)	234 (100%)	34 (14.5%)	≥ 10% tumor cells with all membrane staining
Li* et al. *[81], 2016	China	137	Median: 59.2	101 (73.7%)	I–IV	Intestinal: 60 (43.8%) Diffuse/mixed: 76 (55.5%)	30 (21.9%)	30 (100%)	≥ 5% tumor cells with membrane staining
Ma C.* et al.* [82], 2016	USA	44	Mean: 73	25 (56.8%)	I–IV	GCLS: 16 (36.4%)Adenocarcinoma: 25 (56.8%)Other: 3 (6.8%)	7 (15.9%)	7 (100%)	≥ 5% tumor cells with membrane staining

*EBV* Epstein-Barr virus, *GCSL* gastric cancer with lymphoid stroma, *TC* tumor cells, *IRS* immune reactive score, *CPS* combined positive score, *ROC* receiver operating characteristics, *NR* Not reported

*subgroup of 146 patients with 7 EBV positive

Histologic characterization was heterogeneous among studies. While most authors used Lauren’s classification, a few described tumor’s histology by the *World Health Organization*’s (WHO) criteria. Six studies described the inclusion of GCLS [46, 51, 57, 62, 64, 65], and in 2 studies all included cases corresponded to this histological subtype [62, 65]. In the latter publications, most patients presented with early-stage GC, with stage III and IV corresponding to 19.0% [62] and 9.7% [65]. One author compared GCLS and non-GCLS according to staging, observing a higher rate of pT3-4 disease in GCLS tumors (75.0% vs. 50.0%, *p* = 0.04), but lower rates of node positivity (62.5% vs. 88.9%, *p* = 0.01) and distant metastasis (4.2% vs. 13.0%, *p* = 0.02) [51].

EBV expression in tumor cells was assessed by in situ hybridization (ISH) using an EBV-encoded RNA (EBER) probe in all studies, with a variability of 1.2% to 100% along the included publications. All but one study assessed PD-L1 expression by immunohistochemistry (IHC), nevertheless there was a variety of criteria for PD-L1 positivity on tumor cells: most studies addressed the proportion of tumor cells with membrane staining, with cut-off values ranging from ≥ 1 to ≥ 25%; 6 studies used criteria based on immune reactive score (IRS) [43, 51, 64, 66, 76, 77]; and 5 integrated PD-L1 staining on immune cells on their positivity criteria (for example, by determining the CPS) [42, 46, 48, 49, 68]. Considering the high variability of criteria, PD-L1 expression rate in GC varied among studies, ranging from 3.1% to 85.5%. Further information on PD-L1 staining methods is available on Supplementary Table 1. PD-L1 expression in EBV GC was also variable among publications, ranging from 0.0% to 100%.

### Association of PD-L1 and EBV expression

**Fig. 2:**
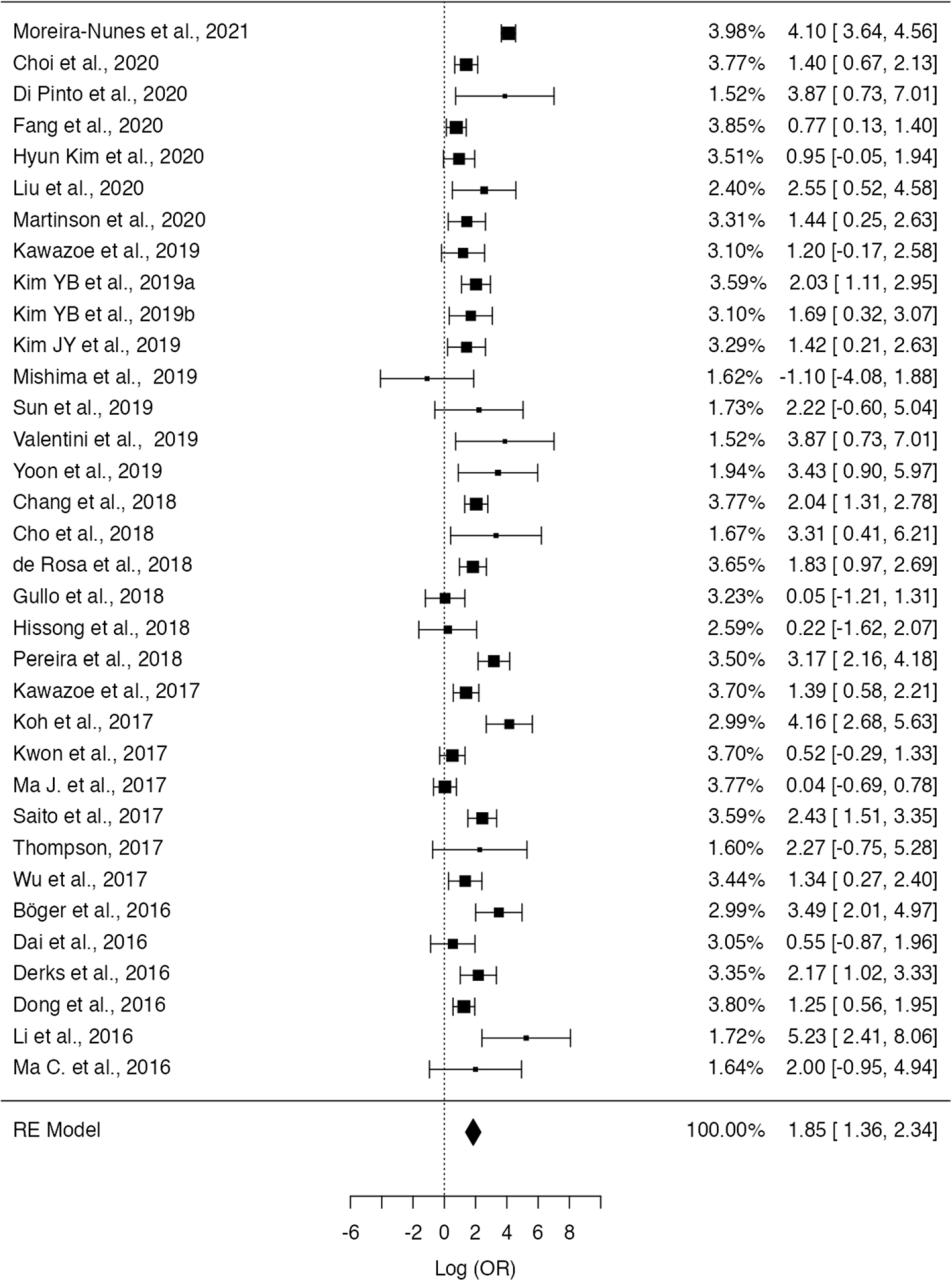
Forest-plot describing the association between EVB and PD-L1 expression. Logarithm for OR is represented in the forest-plot

**Fig. 3:**
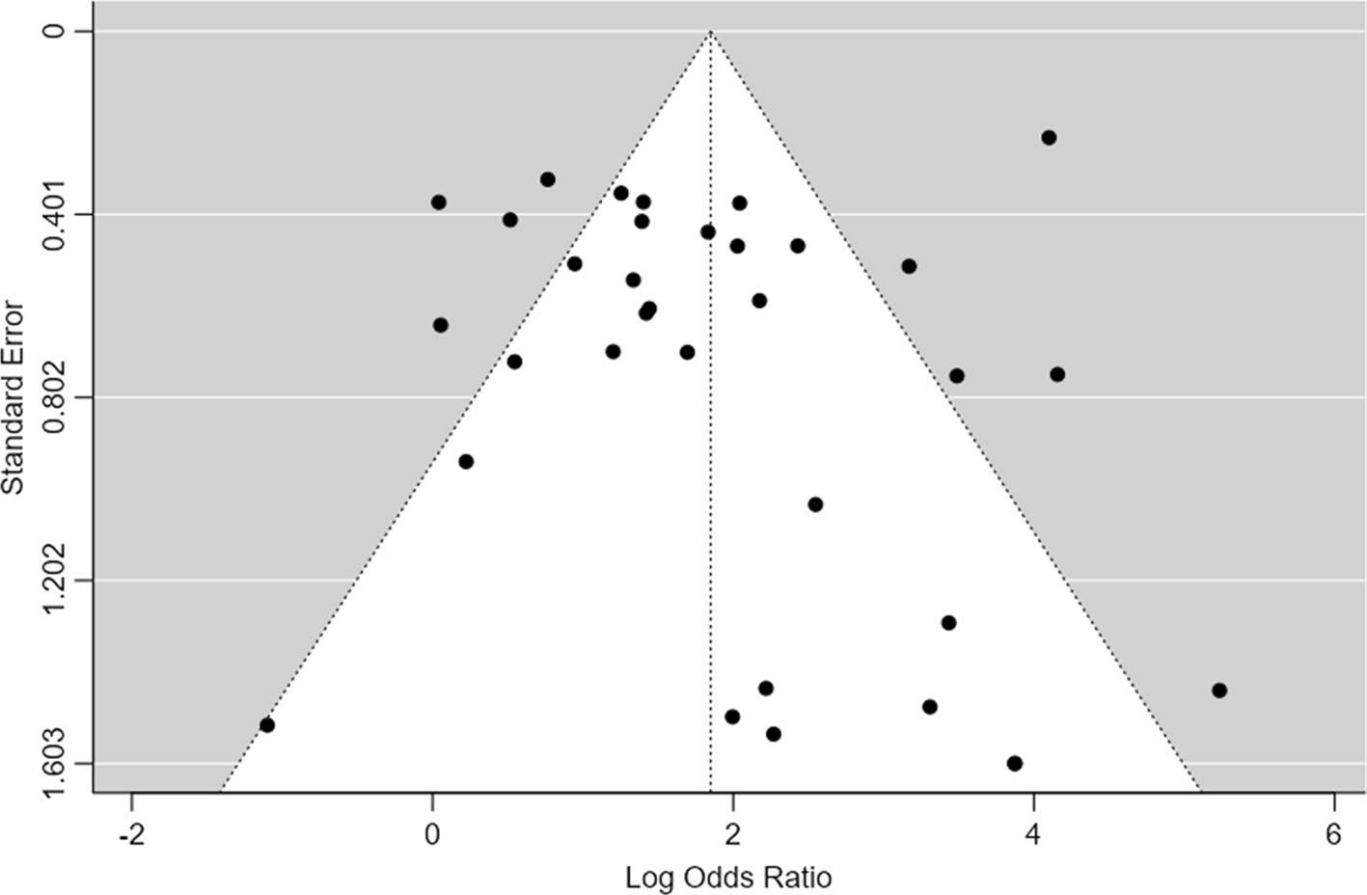
Funnel-plot on the log(OR) of the included patient sets

Thirty-three studies (corresponding to 34 sets) described the frequency of PD-L1 positivity in both EBV-positive (EBVaGC) and negative GC and were included in the meta-analysis. Fig.  2 shows the forest plot for the included patient sets. Overall, we observed that a significantly higher expression of PD-L1 in EBVaGC, with 97% of the OR estimates over 1.00. The estimated pooled OR obtained was of 6.36 (95% confidence interval (CI) [3.91–10.3], Z = 7.45, *p* < 0.001) with a significantly high heterogeneity (τ^2^ = 1.49, I^2^ = 83.7%, Q(33) = 202, *p* < 0.001). No significant publication bias was identified, either by visual inspection or funnel-plot asymmetry regression test (Z = 1.42, *p* = 0.16)—Fig. 3.

### Proportion of PD-L1 expression in EBVaGC

**Fig. 4:**
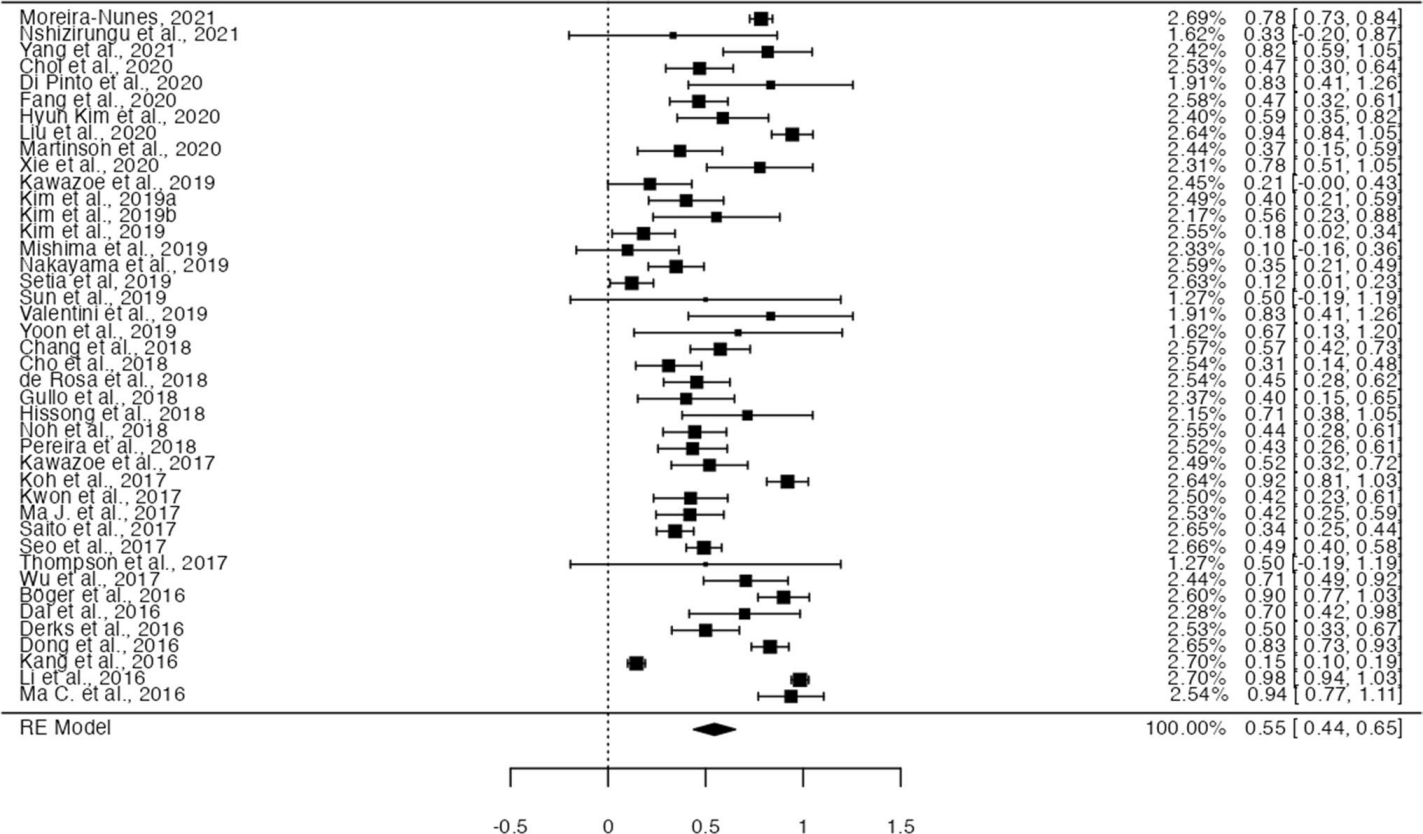
Forest-plot describing the proportion of PD-L1 positivity in EBV positive GC

**Fig. 5:**
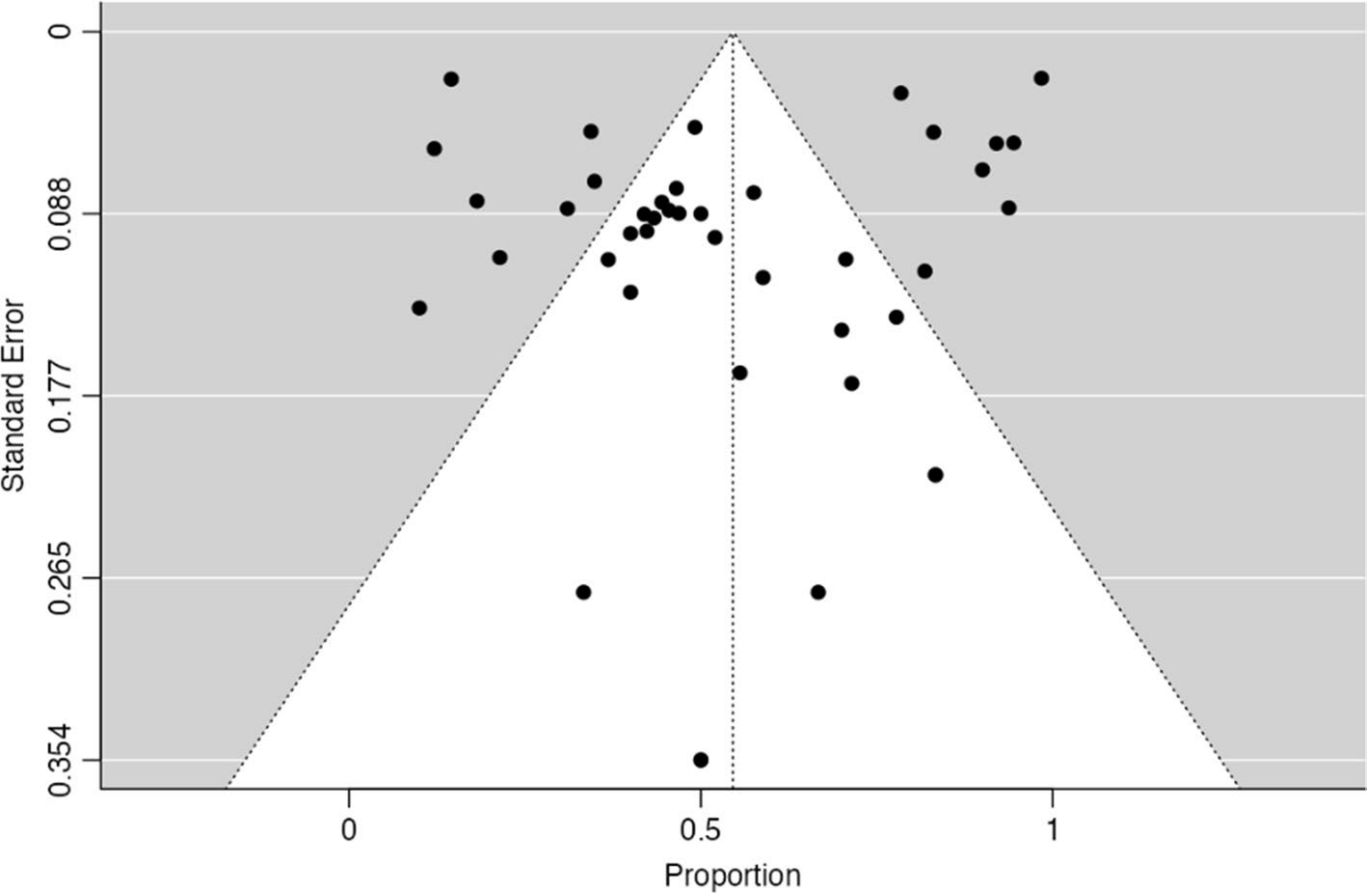
Funnel-plot on the proportion of the included patient sets

Forty-one studies (corresponding to 42 sets of patients) described the frequency of PD-L1 positivity in EBVaGC, allowing their inclusion in a meta-analysis for proportions. Fig.  4 shows the forest plot for the included sets, with a pooled rate of PD-L1 positivity in EBVaGC of 54.6% (95% CI [43.8–65.3%], *p* < 0.001). Data showed a significantly high heterogeneity (τ^2^ = 0.11, I^2^ = 96.2%, Q(41) = 1073, *p* < 0.001); nevertheless, no significant publication bias was identified, either by visual inspection or funnel-plot asymmetry regression test (Z = 0.027, *p* = 0.98)—Fig. 5**.**

### Association of EBV with GCLS

**Fig. 6:**
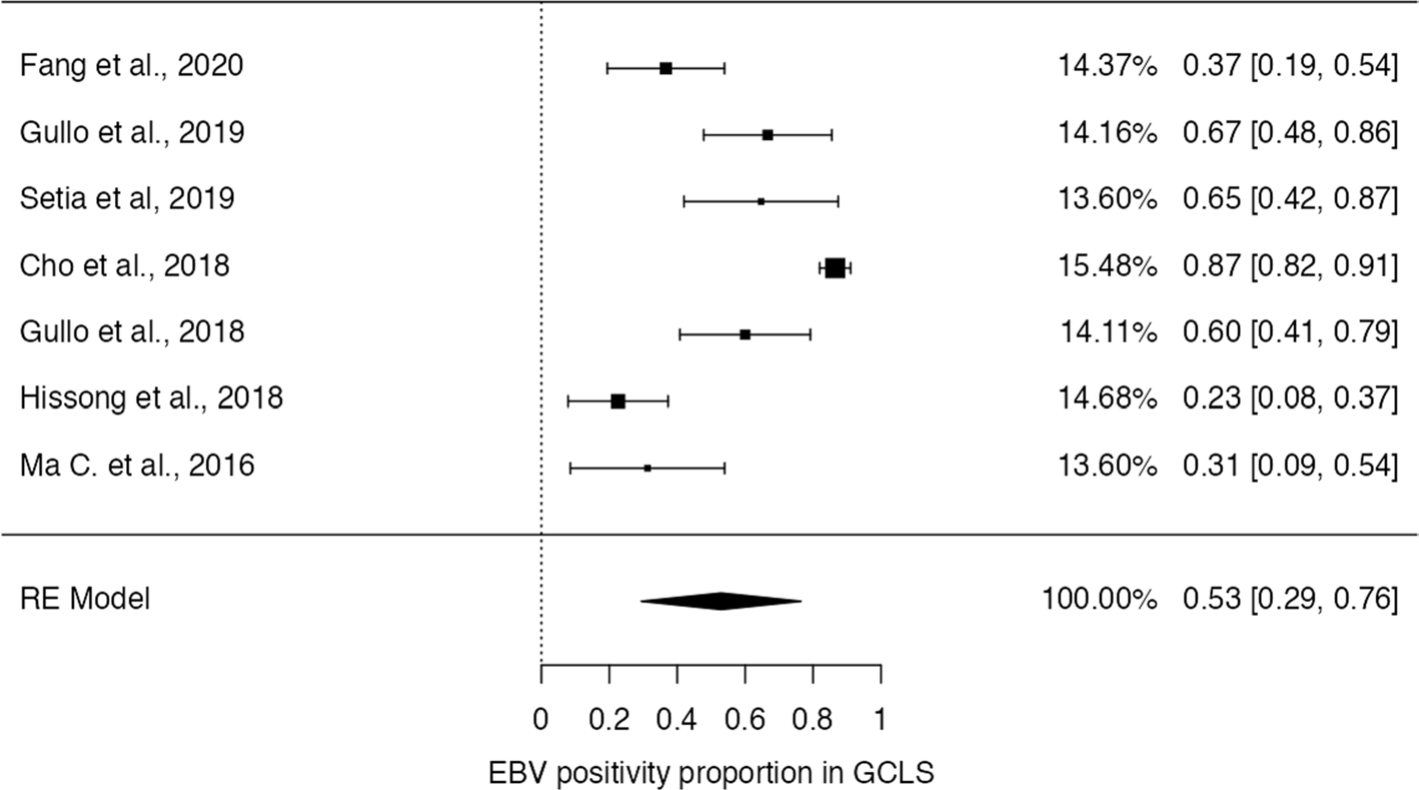
Forest-plot describing the proportion of EBV positivity in GCLS

**Fig. 7:**
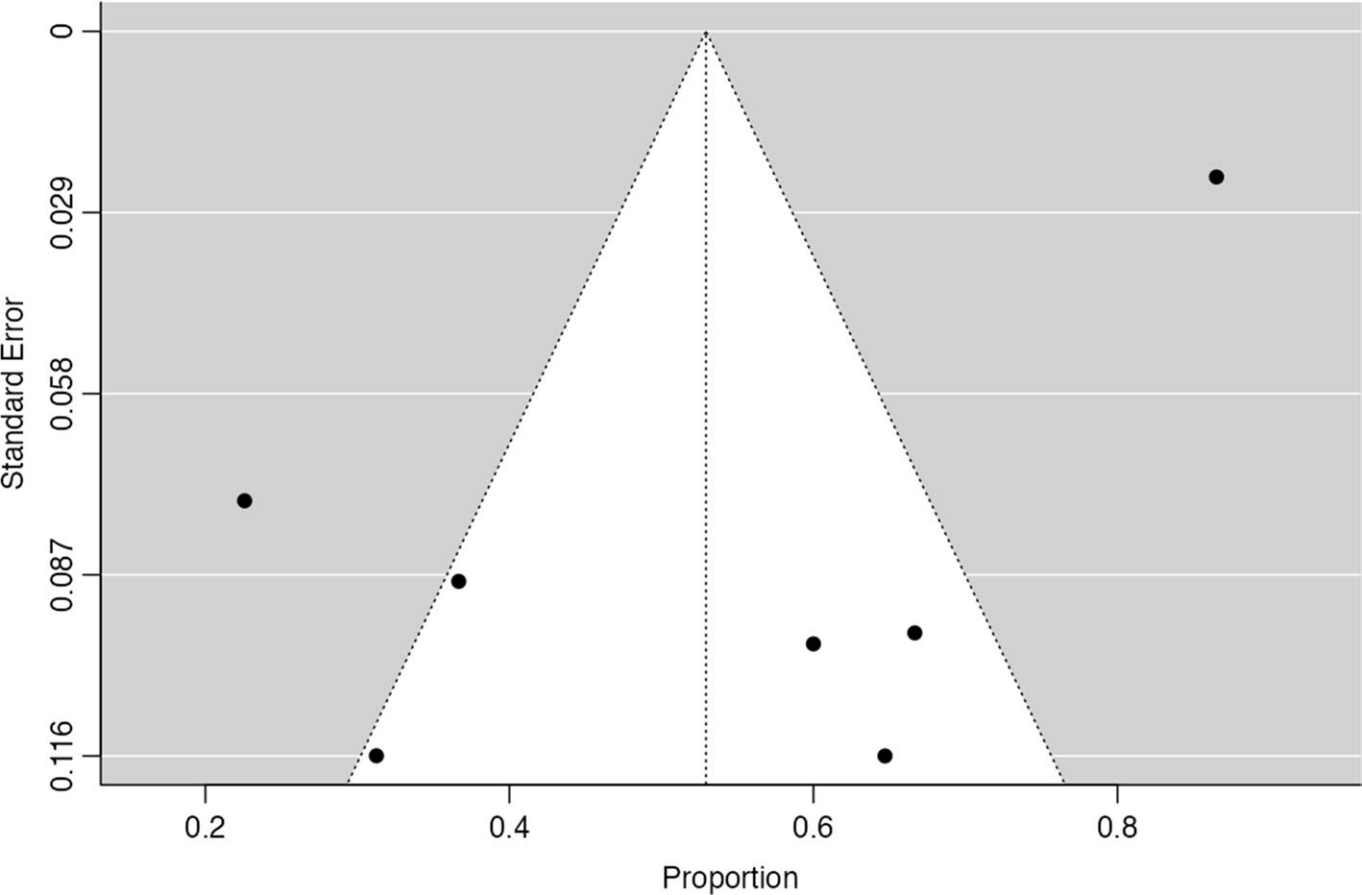
Funnel-plot on the proportion of EBV positivity rate on GCLS

A total of 7 studies described the proportion of EBV in GCLS, with a pooled rate of 52.9% (95% CI [29.4–76.5%], *p* < 0.001) and a significantly high heterogeneity between studies (τ^2^ = 0.30, I^2^ = 94.6%, Q(6) = 110, *p* < 0.001) – Fig. 6. Fig.  7 shows the funnel plot for the raw proportions of the patient sets, where no significant asymmetry is observed, both by visual inspection and funnel-plot asymmetry regression test (Z = -1.05, *p* = 0.30).

**Fig. 8:**
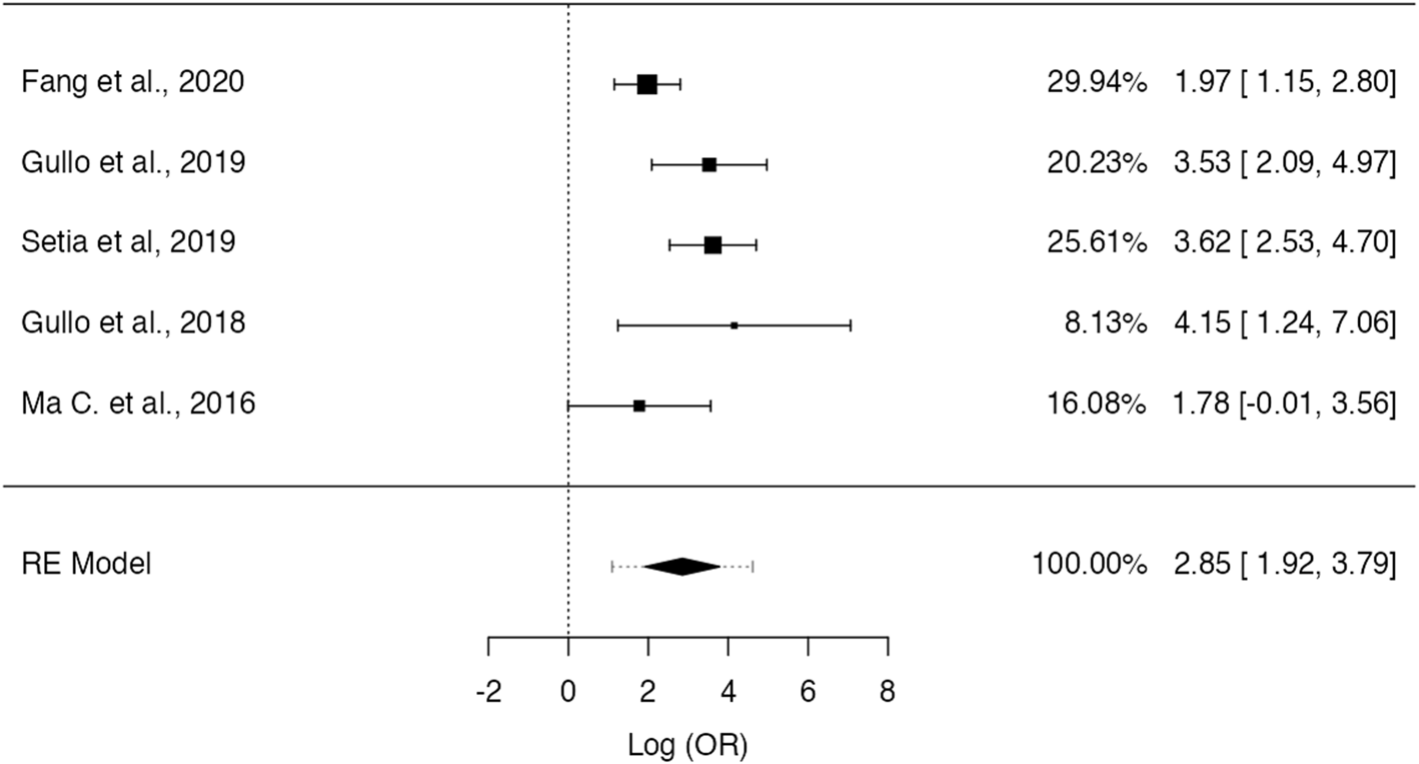
Forest-plot describing the association between EVB and GCLS. Logarithm for OR is represented in the forest-plot

**Fig. 9:**
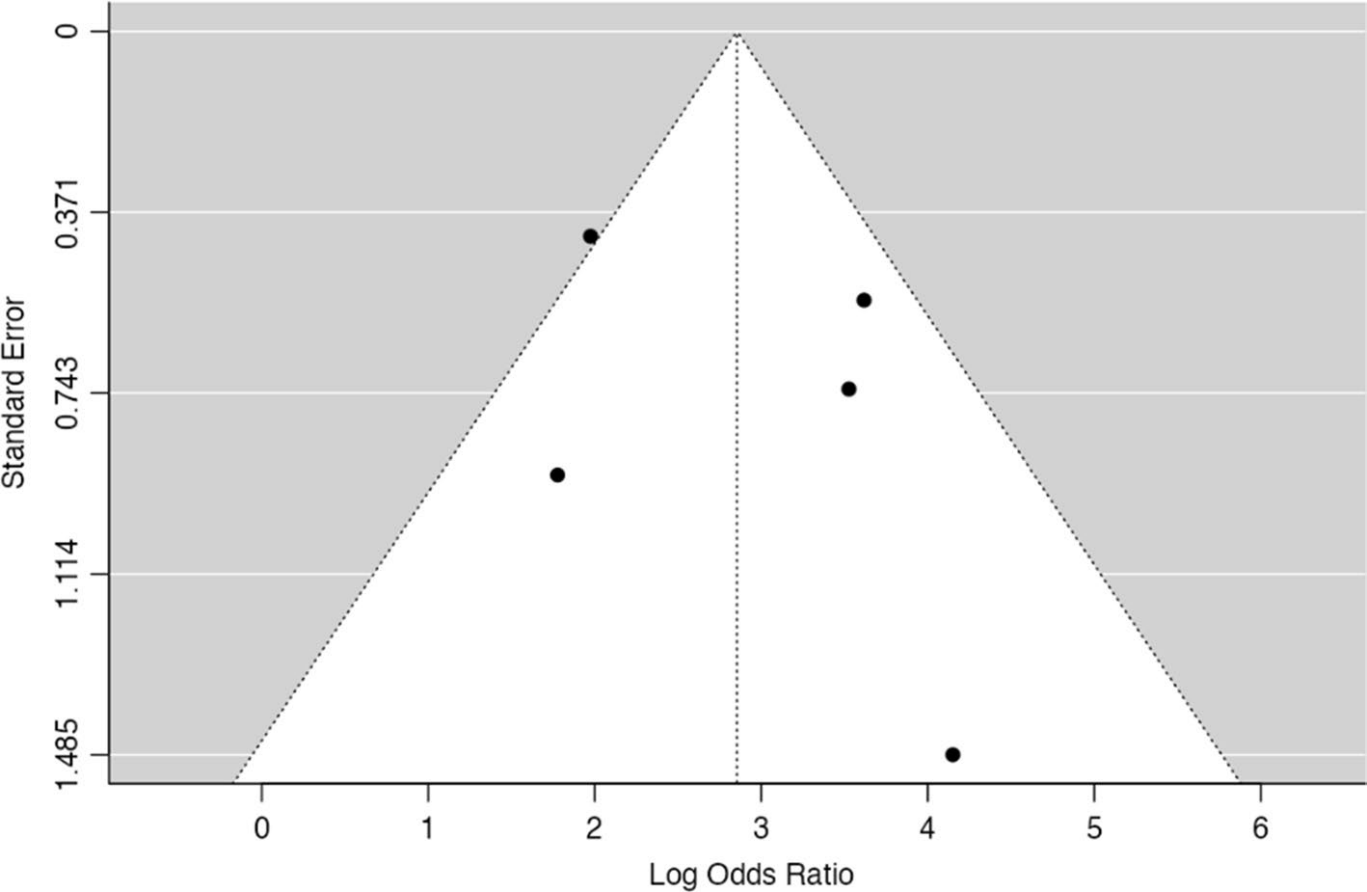
Funnel-plot on the log(OR) related to EBV expression on GCLS

Five publications also described the frequency of EBV positivity in non-GCLS tumors, allowing a metanalysis on OR. Fig.  8 shows the forest plot for these studies, showing a significantly higher expression of EBV in GCLS, with an estimated pooled OR of 17.4 (95% CI [6.83–44.1], Z = 6.00, *p* < 0.001). No significant heterogeneity was observed (τ^2^ = 0.58, I^2^ = 55.4%, Q(4) = 8.98, *p* = 0.062). Fig.  9 shows the funnel plot for the log(OR) of the included studies and no significant publication bias was identified (Z = 0.66, *p* = 0.51).

### Association of PD-L1 with GCLS

**Fig. 10:**
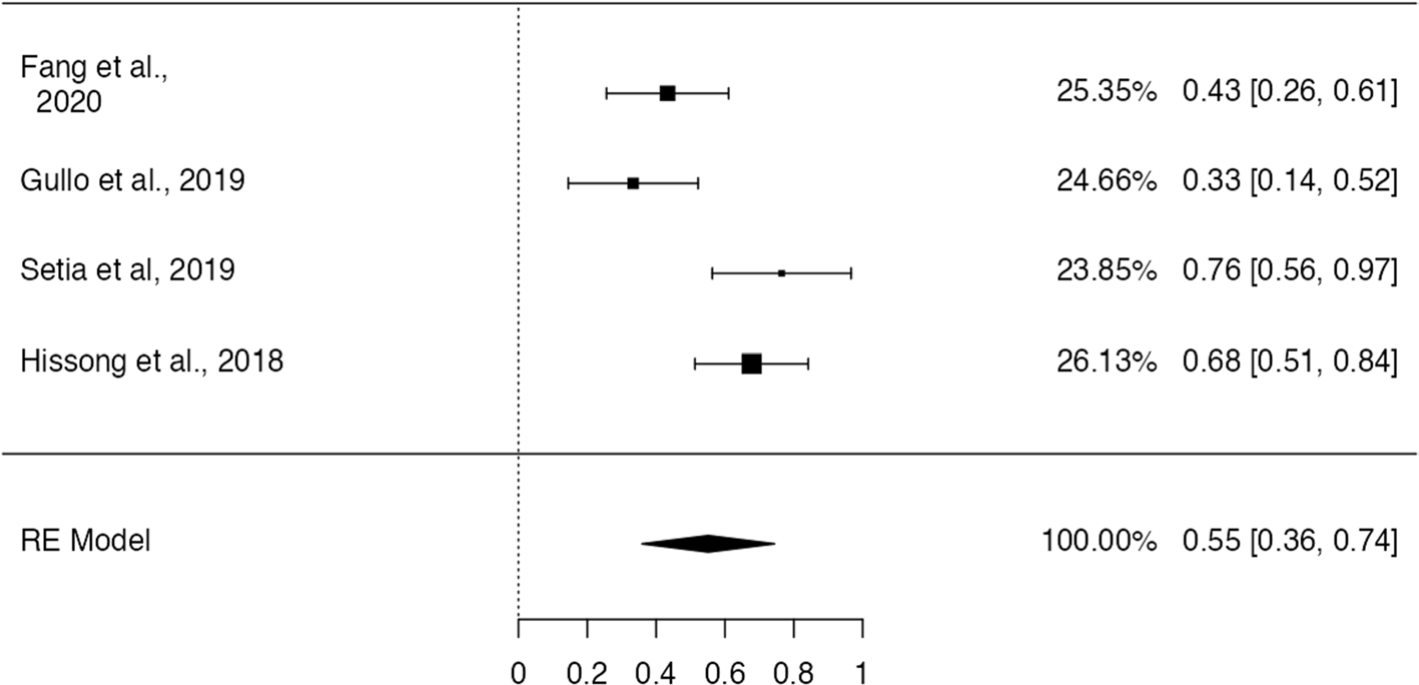
Forest-plot describing the proportion of PD-L1 positivity in GCLS

**Fig. 11:**
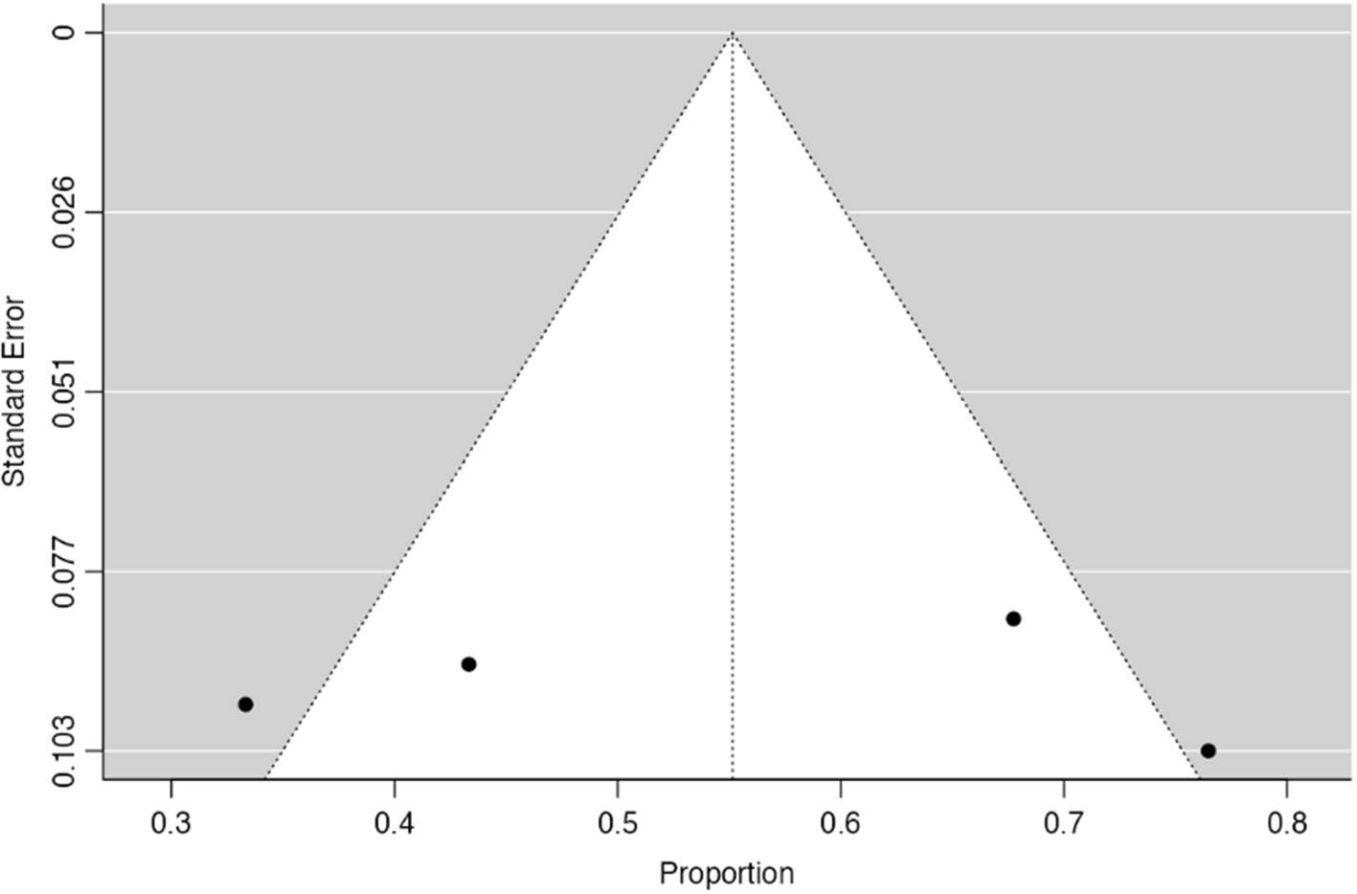
Funnel-plot on the proportion of PD-L1 positivity rate on GCLS

A total of 4 publications addresses the proportion of GCLS tumors which expressed PD-L1. A pooled rate of 55.2% was estimated, with a 95% CI of [35.9–74.4%] (*p* < 0.001), showing a significant heterogeneity (τ^2^ = 0.17, I^2^ = 77.6%, Q(3) = 13.4, *p* = 0.004). Fig.  10 shows the forest-plot regarding proportion estimation. The funnel plot for the raw proportions showed no significant asymmetry by visual inspection and funnel-plot asymmetry regression test (Z = 0.14, *p* = 0.89) —Fig. 11.

**Fig. 12:**
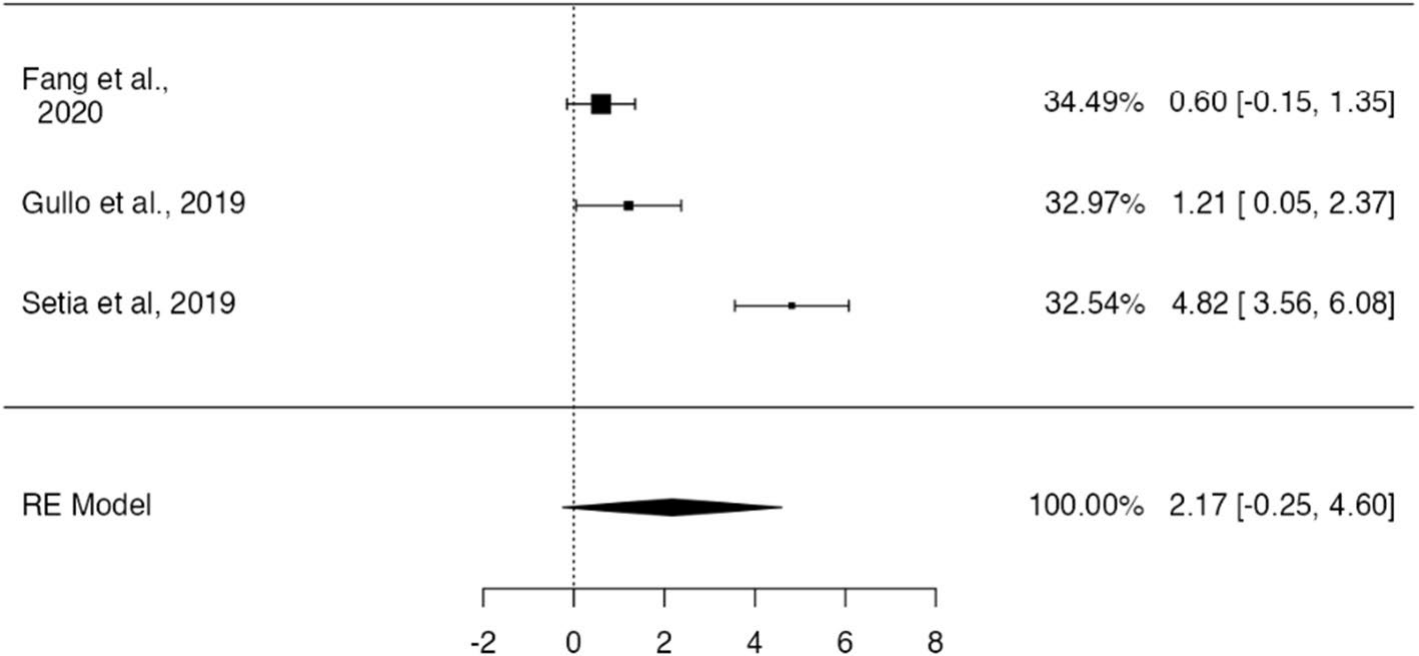
Forest-plot describing the association between PD-L1 and GCLS. Logarithm for OR is represented in the forest-plot

**Fig. 13:**
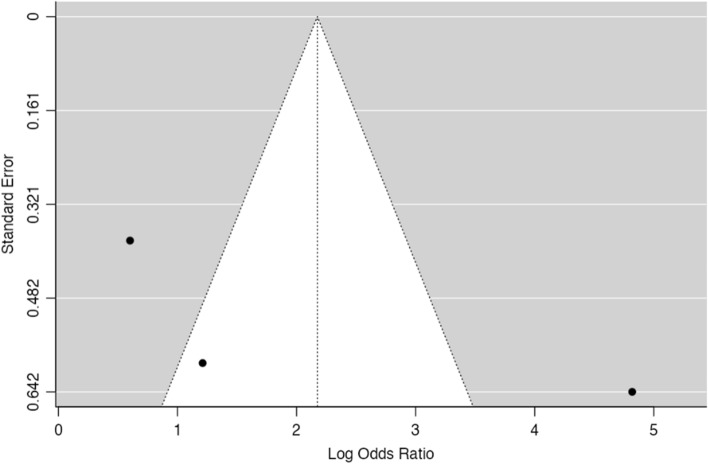
Funnel-plot on the log(OR) related to PD-L1 expression on GCLS

Of these 4 publications, 3 described PD-L1 positivity for both GCLS and non-GCLS gastric tumors. The random-effect model showed a pooled OR of 8.80, although not significantly different from 1 (95% CI [0.78− 99.2], *p* = 0.079, Fig. 12). A significant heterogeneity was observed for this model (τ^2^ = 2.07, I^2^ = 93.8%, Q(2) = 32.3, *p* < 0.001), with no significant asymmetry by visual inspection and funnel-plot asymmetry regression test (Z = 1.12, *p* = 0.26)—Fig. 13.

## Dicussion

GC has a high impact in populations, since it is frequently diagnosed at advanced stages and, therefore, the potential for successful treatment is limited. Over the past few years, several trials have been carried out to develop new therapeutic strategies. The knowledge of the host immune system regulation and its role in cancer development are one of the current focus of anticancer drug research, acting on the immune checkpoints seems, especially with the aim to sustain or increase the activity of the immune system to destroy the tumor cells. Immunotherapy has established a firm position in the treatment of different solid tumors such melanoma, lung cancer and clear-cell renal cancer, but its role in the treatment of GC is much less defined.

Since TCGA proposed a molecular classification of GC that it has been increasing the search for potential biomarkers for the treatment of the distinct subtypes. Despite EBVaGC represents only around 10% of all GC, the evidence of specific genomic signatures pointed to development of targeted therapies [3, 22–25]. Literature has been supporting the idea that EBVaGC has a distinctive genomic profile including high levels of programmed death ligands 1 and 2 (PD-L1/2) expression, which can be used as a surrogate marker for immunotherapy [2, 10, 14–19]. In this systematic review and meta-analysis, we revealed a significantly higher pooled expression of PD-L1 in EBV associated GC. To the best of our knowledge, this is the first systematic review and meta-analysis specifically addressing this issue.

As previously mentioned, PD-L1 is expressed in some tumor cells and binds to the PD-1 receptor in T lymphocytes, inhibiting their ability to initiate an immune response against cancer cells. An overexpression of this marker, either on tumor cells or on tumor infiltrating lymphocytes, is associated with better response rates and overall survival (OS). In fact, there is evidence of better response to immunotherapy on patients overexpressing PD-L1 for other primary tumors. For example, KEYNOTE-024 showed a significant benefit of pembrolizumab as first-line monotherapy comparing to platinum-based chemotherapy for advanced non-small cell lung cancer (NSCLC) with PD-L1 expression over 50%, in both response rate and OS [83, 84]. For head and neck squamous cell carcinoma, a phase III trial showed that nivolumab as second-line therapy brings an OS benefit in patients with tumor PD-L1 expression ≥ 1%, compared to standard second-line therapy [85]. Also, KEYNOTE-048 showed a significant advantage of a combination of pembrolizumab with a platinum and fluoropyrimidine based chemotherapy scheme for recurrent or metastatic cancer when CPS (defined as the number of staining tumor cells, macrophages and lymphocytes divided by the total number of tumor cells, and multiplied by 100) is ≥ 20 [86]. Of note, CPS includes all PD-L1 positive cells within the tumor in its determination, which reflects the importance of the tumor microenvironment for the response to anti-PD-1/PD-L1 targeted therapy. In this review, only the immunoreactivity of tumor cells was assessed for meta-analysis since it was the most frequently reported result in the included studies. Although it would be useful to assess the influence of tumor microenvironment in EBVaGC, PD-L1 expression in tumor-related immune cells was heterogeneously described. Also, none of the included studies was designed to assess response rate or survival for EBVaGC expressing PD-L1 treated with anti-PD-1/PD-L1 immunotherapy. It is important to assess the prognostic impact of patient selection for targeted immunotherapy in GC using molecular markers in future studies, since it might improve response rate, patient survival and minimize unneeded side effects.

This review estimated a PD-L1 positivity rate of about 55% in EBV associated GC, although resulting from a significant variation across the analyzed studies. This variation might be related to different histologic types included, since EBV expression is most evident in GCLS and adenocarcinomas showing Crohn’s disease-like lymphoid reaction (87). Particularly, GCLS tumors are frequently associated with EBV, with a positivity rate among this histologic subtype over 80% [21, 62, 88]. In other EBV-associated solid tumors, such as nasopharyngeal carcinomas, tumor PD-L1 expression rate is as high as 70%, but the correlation between PD-L1 expression and survival is unclear [89].

GCLS constitutes a rare subgroup of GC, accounting for about 1–4% of GC [21], composed by packed tumor cells with lymphocytic stomal and tumor infiltration [90]. Different studies have associated this histology with a favorable prognosis [91, 92], with lower frequency of lymph node metastasis [93, 94]. Association of this histological subtype with EBV infection has previously been established [20, 21]. Our meta-analyses confirmed a significantly higher EBV expression on this histologic subtype compared to non-GCLS histology, with a pooled positivity rate of about 53%. This association might account for better prognosis for this histology, since EBV expression is associated with better prognosis [8, 9]. Further studies are necessary to address this issue.

Few studies included in this systematic review reported PD-L1 expression in GCLS. Although a non-significant pooled association between this rare histology and PD-L1 expression was obtained, a pooled proportion of 55% was observed for this histological subtype. Further studies are needed in order to address this particular marker in GCLS, in association with EBV expression and prognosis, as it might help understanding the molecular mechanisms underlying this histology, as well as identifying prognostic markers for targeted therapy.

There are some relevant limitations to the present systematic review. A significant heterogeneity among the included studies was observed, with some studies identifying less than 10 patients with EBVaGC [45, 55, 75], reflecting the prevalence of this molecular subtype among GC, estimated to be 9% [4–7]. The different PD-L1 expression rates might also be influenced by patient related factors, such as age, gender, staging, location and histologic type, for which we were not able to control. Also, methods for assessing PD-L1 expression were heterogeneous among studies, both regarding antibodies used for IHC and positivity criteria. In fact, the cut-off used for positivity ranged from 1% of tumor cells staining for PD-L1 [52, 67, 69] to 25% [62]; other studies considered a combined analysis of tumor cell and macrophage staining [57], CPS-related criteria [68], or an immunoreactive scoring system, accounting for both percentage of immunoreactive cells and staining intensity [51, 64]. This might reflect the wide range of PD-L1 positivity rate in EBV positive GC revealed in this review.

Although publication bias analysis was not significant, we should mention that the selection criteria only included reports written in English, which might limit the number of relevant studies included in this review. Also, most studies were originated in Asian countries, reflecting a higher incidence of GC and, therefore, better availability of data [1]. Our conclusions may, therefore, be only applicable to these specific populations and not easily generalized. Another limitation is that most of the included publications were observational and retrospective studies. In fact, patient selection might be compromised in some cases, namely since the control group selection criteria was not always the same as the experimental group, as observed in the study by Sundar, et al. [68]].

In conclusion, patients with EBVaGC tend to show a higher PD-L1 expression, which enhances EBV positivity as a promising marker for patient selection for anti-PD-1/PD-L1 targeted therapy. Particularly GCLS histology showed a higher EBV expression, although an association analysis with PD-L1 expression was not possible. Still, there is a need for uniform criteria for PD-L1 positivity, and further large-scale prospective studies are needed to validate these findings and assess their prognostic significance.

## Supplementary Information

Additional file1 (DOCX 99 KB)

Additional file2 (DOCX 43 KB)

## Data Availability

Not applicable.

## Abbreviations

CAR: Chimeric antigen receptor
CI: Confidence interval
CPS: Combined positive score
EBV: Epstein-Barr virus
EBER: Epstein-Barr virus encoded RNA
FDA: Food and drug administration
GC: Gastric cancer
GCLS: Gastric cancer with lymphoid stroma
HER-2: Human epidermal growth factor receptor 2
IHC: Immunohistochemistry
IRS: Immune reactive score
ISH: In situ hybridization
MMR: Mismatch repair
MSI-H: High microsatellite instability
NOS: Newcastle–Ottawa scale
NSCLC: Non-small cell lung cancer
OR: Odds ratio
OS: Overall survival
PD-1: Programmed cell death protein 1
PD-L1: Programmed cell death protein ligand 1
PRISMA: Preferred reporting items for systematic reviews and meta-analyses
RNA: Ribonucleic acid
ROC: Receiver operating characteristics
TC: Tumor cell
TCGA: The cancer genome atlas
VEGF: Vascular endothelial growth factor
WHO: World Health Organization
