# Supplementary material for: PD-L1 expression in EBV associated gastric cancer: a systematic review and meta-analysis
Source: Discov Oncol. 2022 Mar 22;13:19. doi: 10.1007/s12672-022-00479-0 (PMC8941030; doi:10.1007/s12672-022-00479-0)
Supplement: Supplementary file 1 — Additional file1 (DOCX 99 KB) [file 12672_2022_479_MOESM1_ESM.docx]

### Supplementary Table 1: PD-L1 determination methods.

| **Authors (reference number), year** | **Method** | **Clone / Reference** | **Company** | **Host** | **Monoclonal** | **Dilution** | **PD-L1 positivity criteria** |
| --- | --- | --- | --- | --- | --- | --- | --- |
| Moreira-Nunes *et al*. (41), 2021 | SDS-PAGE | MIH1 | Thermo Fisher Scientific, Carlsbad, CA, USA | ND | ND | ND | Comparison to non-tumor controls (higher vs. lower) |
| Nshizirungu *et al.* (42), 2021 | IHC | 22C3 | ND | Mouse | Yes | ND | CPS > 1 |
| Yang *et al.* (43), 2021 | IHC | E1L3N | Cell Signaling Technology, Cambridge, UK | ND | ND | 1:200 | IRS > 2 |
| Choi *et al*. (44), 2020 | IHCBenchMark Ultra autostainer | E1L3N | Cell Signalling Technology, Danvers, MA, USA | Rabbit | Yes | 1:30 | Any membrane staining in tumor cells |
| Di Pinto *et al*. (45), 2020 | IHCDako autostainer, Glostrup, Denmark | E1L3N | Cell Signalling Technology, Danvers, MA, USA | Rabbit | Yes | 1:800 | ≥ 5% tumor cells with membrane staining |
| Fang *et al*. (46), 2020 | IHC Dako ASL48 platform | 22C3 | ND | ND | ND | ND | CPS ≥ 1 |
| Hyun Kim *et al*. (47), 2020 | IHC | SP263 | Ventana Medical Systems Inc., Tucson, AZ | ND | ND | ND | ≥ 1% tumor cells with membrane staining |
| Liu *et al*. (48), 2020 | IHCDako Autostainer Link 48 system | 22C3 pahrmDx | Agilent Technologies, Carpinteria, CA, USA | Rabbit | Yes | ND | CPS ≥ 1 |
| Martinson *et al*. (49), 2020 | IHC | 22C3 pharmDx | Agilent Technologies, Carpinteria, CA, USA | Rabbit | Yes | ND | CPS ≥ 1 |
| Xie *et al*. (50), 2020 | IHC | 22C3 pharmDx | Agilent Technologies, Carpinteria, CA, USA | ND | ND | ND | ≥ 5% tumor cells with membrane staining |
| Gullo *et al.* (51), 2019 | IHC | E1L3N | Cell Signalling Technology, Danvers, MA, USA | Rabbit | Yes | 1:1000 | IRS > 2 |
| Kawazoe *et al.* (52), 2019 | IHCDako autostainer, Glostrup, Denmark | 22C3 pharmDx | Agilent Technologies, Carpinteria, CA, USA | Rabbit | Yes | ND | ≥ 1% tumor cells with membrane staining |
| Kim YB *et al.* (53), 2019, | IHC | SP263 | Ventana Medical Systems Inc., Tucson, AZ | Rabbit | Yes | ND | ≥ 5% tumor cells with membrane staining |
| Kim JY *et al*. (54), 2019 | IHC | E1L3N | Cell Signalling Technology, Danvers, MA, USA | Rabbit | Yes | 1:200 | > 5% tumor cells with membrane staining |
| Mishima *et al*. (55), 2019 | IHC | SP142SP263 | Ventana Medical Systems Inc., Tucson, AZ | Rabbit | Yes | ND | ≥ 5% tumor cells with membrane staining |
| Nakayama *et al*. (56), 2019 | IHC | E1L3N | Cell Signalling Technology, Danvers, MA, USA | Rabbit | Yes | 1:200 | ≥ 5% tumor cells with membrane staining |
| Setia *et al*, (57), 2019 | IHC | E1L3N | Cell Signalling Technology, Danvers, MA, USA | Rabbit | Yes | 1:200 | Any membrane staining in tumor or macrophages |
| Sun *et al*. (58), 2019 | IHC | E1L3N | Cell Signalling Technology, Danvers, MA, USA | Rabbit | Yes | 1:200 | ≥ 1% tumor cells with membrane staining |
| Valentini *et al*. (59), 2019 | IHC | E1L3N | Cell Signalling Technology, Danvers, MA, USA | Rabbit | Yes | 1:800 | ≥ 5% tumor cells with membrane staining |
| Yoon *et al*. (60), 2019 | IHC | 22C3 | Ventana Medical Systems Inc., Tucson, AZ | ND | ND | ND | > 1% tumor cells with membrane staining |
| Chang *et al.* (61), 2018 | IHC | SP142 | Ventana Medical Systems Inc., Tucson, AZ | Rabbit | Yes | ND | PD-L1 ratio > .136441(automated method) |
| Cho *et al.* (62), 2018 | IHC | E1L3N | Cell Signalling Technology, Danvers, MA, USA | Rabbit | Yes | 1:100 | ≥ 25% tumor cells with membrane staining |
| de Rosa *et al*. (63), 2018 | IHC | SP142 | Spring Bioscience, CA, USA | Rabbit | Yes | 1:100 | ≥5% membrane staining, any intensity |
| Gullo *et al.* (64), 2018 | IHC | E1L3N | Cell Signalling Technology, Danvers, MA, USA | Rabbit | Yes | 1:1000 | IRS ≥ 2 |
| Hissong *et al*. (65), 2018 | IHC | SP142 | Spring Bioscience, CA, USA | Rabbit | Yes | 1:100 | Any membrane staining in tumor cells |
| Noh *et al*. (66), 2018 | IHC | SP263 | Roche Diagnostics, Tucson, USA | ND | ND | ND | IRS ≥ 2 |
| Pereira *et al.* (67), 2018 | IHC | 28-8 | Abcam, Cambridge, UK | Rabbit | Yes | 1:50 | ≥ 1% tumor cells with membrane staining |
| Sundar *et al.* (68), 2018 | IHC | 22C3 pharmDx | Agilent Technologies, Carpinteria, CA, USA | Rabbit | Yes | ND | CPS > 1 or > 5 |
| Kawazoe *et al.* (69), 2017 | IHCDako autostainer, Glostrup, Denmark | SP142 | Ventana Medical Systems Inc., Tucson, AZ | Rabbit | Yes | ND | ≥ 1% tumor cells with membrane staining |
| Koh *et al*. (70), 2017 | IHC | E1L3N | Cell Signalling Technology, Danvers, MA, USA | Rabbit | Yes | E1L3N | ≥ 5% tumor cells with membrane staining |

### (continues on next page).

### Supplementary Table 1: PD-L1 determination methods.

| **Authors (reference number), year** | **Method** | **Clone / Reference** | **Company** | **Host** | **Monoclonal** | **Dilution** | **PD-L1 positivity criteria** |
| --- | --- | --- | --- | --- | --- | --- | --- |
| Kwon *et al*. (71), 2017 | IHC | SP142 | Ventana Medical Systems Inc., Tucson, AZ | Rabbit | Yes | 1:25 | > 10% tumor cells with membrane staining |
| Ma J. *et al*. (72), 2017 | IHC | ND | Abcam, Cambridge, MA | ND | ND | ND | ≥5% membranous expression were considered positive. |
| Saito *et al.* (73), 2017 | IHC | E1L3N | Cell Signalling Technology, Danvers, MA, USA | Rabbit | Yes | 1:200 | > 5% tumor cells with membrane staining |
| Seo *et al*. (74), 2017 | IHC | E1L3N | Cell Signalling Technology, Danvers, MA, USA | Rabbit | Yes | 1:100 | ≥1% tumor cells with moderate or strong staining |
| Thompson *et al.* (75), 2017 | IHC | 5H1 | ND | Mouse | Yes | ND | ≥ 5% tumor cells with membrane staining |
| Wu *et al*. (76), 2017 | IHC | E1L3N | Cell Signaling Technology, Cambridge, UK | ND | ND | 1:200 | IRS > 2 |
| Böger *et al.* (77), 2016 | IHC | E1L3N | Cell Signalling Technology, Danvers, MA, USA | Rabbit | Yes | 1:75 | IRS > 2 |
| Dai *et al.* (78), 2016 | IHCDako autostainer Plus Link 48 | MKP1A07310 | Merck-Serono, Darmstadt, Germany | Rabbit | Yes | ND | ≥ 5% tumor cells with membrane staining or ≥ 1+ intensity |
| Derks *et al.* (19), 2016 | IHC | 405.9A11 | ND | Mouse | ND | ND | ≥ 5% tumor cells with membrane staining |
| Dong *et al.* (79), 2016 | IHCStep EnVision IHC procedure, Dako, Glostrup, Denmark | ab58810 | Abcam, Cambridge, MA | Rabbit | No | 1:100 | Cut-off determined for this sample using a ROC curve |
| Kang *et al.* (80), 2016 | IHC | 13684S | Cell Signaling, Beverly, MA, USA | Rabbit | Yes | 1:100 | ≥ 10% tumor cells with all membrane staining |
| Li *et al.* (81), 2016 | IHC | SP142 | Spring Bioscience, CA, USA | Mouse | Yes | 1:100 | ≥ 5% tumor cells with membrane staining |
| Ma C. *et al.* (82), 2016 | IHC | SP263 | Ventana Medical Systems Inc., Tucson, AZ | Rabbit | Yes | ND | ≥ 5% tumor cells with membrane staining |

### PD-L1: programmed cell death protein ligand 1, IHC: immunohistochemistry, IRS: immune reactive score, CPS: combined positive score, ROC: receiver operating characteristics, ND: not described.
