# Supplementary material for: PD-L1 expression in EBV associated gastric cancer: a systematic review and meta-analysis
Source: Discov Oncol. 2022 Mar 22;13:19. doi: 10.1007/s12672-022-00479-0 (PMC8941030; doi:10.1007/s12672-022-00479-0)
Supplement: Supplementary file 2 — Additional file2 (DOCX 43 KB) [file 12672_2022_479_MOESM2_ESM.docx]

**Identification of studies via databases and registers**

Records removed *before screening*:

Duplicate records removed (n = 292)

Records identified from:

PubMed^®^ (n = 148)

EMBASE^®^ (n = 261)

Web of Science^®^ (n = 167)

**Identification**

Records screened

(n = 284)

Records excluded

(n = 220)

**Screening**

Reports excluded:

Incomplete data on PD-L1 or EBV (n = 17)

Patient overlap with other publications (n = 3)

Study protocol (n = 1)

Reports assessed for eligibility

(n = 64)

Studies included in review

(n = 43)

**Included**
